# The genetic architecture of leaf stable carbon isotope composition in *Zea mays* and the effect of transpiration efficiency on leaf elemental accumulation

**DOI:** 10.1093/g3journal/jkab222

**Published:** 2021-07-14

**Authors:** Crystal A Sorgini, Lucas M Roberts, Madsen Sullivan, Asaph B Cousins, Ivan Baxter, Anthony J Studer

**Affiliations:** 1 Department of Crop Sciences, University of Illinois, Urbana, IL 61801, USA; 2 School of Biological Sciences, Washington State University, Pullman, WA 99164, USA; 3 Donald Danforth Plant Science Center, St. Louis, MO 63132, USA

**Keywords:** *Zea mays*, carbon isotopes, transpiration efficiency, specific leaf area, ionomics

## Abstract

With increased demand on freshwater resources for agriculture, it is imperative that more water-use efficient crops are developed. Leaf stable carbon isotope composition, δ^13^C, is a proxy for transpiration efficiency and a possible tool for breeders, but the underlying mechanisms effecting δ^13^C in C_4_ plants are not known. It has been suggested that differences in specific leaf area (SLA), which potentially reflects variation in internal CO_2_ diffusion, can impact leaf δ^13^C. Furthermore, although it is known that water movement is important for elemental uptake, it is not clear how manipulation of transpiration for increased water-use efficiency may impact nutrient accumulation. Here, we characterize the genetic architecture of leaf δ^13^C and test its relationship to SLA and the ionome in five populations of maize. Five significant QTL for leaf δ^13^C were identified, including novel QTL as well as some that were identified previously in maize kernels. One of the QTL regions contains an Erecta-like gene, the ortholog of which has been shown to regulate transpiration efficiency and leaf δ^13^C in *Arabidopsis*. QTL for δ^13^C were located in the same general chromosome region, but slightly shifted, when comparing data from two different years. Our data does not support a relationship between δ^13^C and SLA, and of the 19 elements analyzed, only a weak correlation between molybdenum and δ^13^C was detected. Together these data add to the genetic understanding of leaf δ^13^C in maize and suggest that improvements to plant water use may be possible without significantly influencing elemental homeostasis.

## Introduction

The impacts of global population growth and climate change on natural resources indicate that the future of food security will depend on increasing both the productivity and sustainability of agriculture systems (National Academies of Sciences, Engineering, and Medicine 2018). Improving crop water-use efficiency (WUE) would ameliorate the effects of the increasing frequency and severity of droughts (Sheffield and Wood 2008; Chapman *et al.* 2012; Leakey 2019) . Agronomic WUE can be defined as the amount of yield, whether grain or biomass, produced per the total amount of water utilized by the crop (Condon *et al.* 2004). Many factors can affect WUE including transpirational water loss through the stomatal pores on the leaf’s surface. In C_3_ plants the amount of carbon available for assimilation is limited by stomatal and mesophyll conductances to CO_2_ (Flexas *et al.* 2016) and therefore correlated to the rate of transpiration. For example, yield was shown to be positively associated with cumulative transpiration in soybean (Purcell *et al.* 2007), and higher net carbon assimilation was accompanied by higher transpiration in rice (Adachi *et al.* 2017). However, higher rates of biomass yield do not always correspond to higher transpiration rates in C_4_ plants due to the evolution of the carbon concentrating mechanism. The uncoupling of CO_2_ assimilation and transpiration has been demonstrated in the field and greenhouse-grown maize (Walker 1986; Kolbe *et al.* 2018a). Thus, there is the potential to increase transpiration efficiency, or carbon gain per amount of water transpired, without reducing productivity in C_4_ species (Leakey 2019). A large amount of variation is present in the transpiration rates of C_4_ crop species, including sorghum (Hammer *et al.* 1997), and maize (Bunce 2010), suggesting that existing occurring alleles could be exploited for optimizing WUE.

Although increasing transpiration efficiency provides a strategy to avoid the negative effects of water limitation on plant growth and development (Passioura 1996; Chaves *et al.* 2002; Jaleel *et al.* 2009), there is the possibility of pleiotropic side effects given the fundamental requirement for water movement in plants. A potential impact of reducing transpiration could be a corresponding reduction in the uptake and mobilization of water-soluble nutrients. As water is absorbed by roots, nutrients in solution come in contact with the root surface in a process known as mass flow (Barber *et al.* 1963). Most nutrients are acquired by mass flow, although phosphorus is a notable exception that contacts the root through diffusion (Barber 1962). Reducing transpiration may also affect nutrient uptake facilitated by symbiosis with mycorrhizal fungi (Marschner and Dell 1994). Therefore, the manipulation of basic plant processes such as transpiration for improved WUE must also consider potential impacts on the availability of essential plant nutrients. Essential elements contribute to cellular function in numerous ways including in biochemical reactions as catalytic cofactors, for charge balance in cellular and subcellular compartments, as well as in DNA and protein, the building blocks of life (Baxter 2009). Previous research has shown in the C_4_ plant sorghum that total leaf mineral content is positively correlated with transpiration efficiency (Masle *et al.* 1992). While meta-analyses of high CO_2_ grown plants with reduced transpiration have shown a drastic reduction in nutrient accumulation in several tissues including the leaves and grain of C_3_ crops (McGrath and Lobell 2013), sorghum showed no difference and maize had similar levels of zinc, protein, and phytate, but a decrease in iron accumulation in the grain (Myers *et al.* 2014). Although part of the reduction in nutrient content can be explained by dilution, due to increased growth at high CO_2_, this does not completely account for the observed reduction. An ionomics (high-throughput elemental profiling) approach has been used in maize to assess kernel nutrient content (Baxter *et al.* 2014). A similar ionomics approach in leaf tissue could be used to assess the effect of transpiration on nutrient accumulation in the leaves.

The difficulty and labor-intensive nature of accurately quantifying the amount of water that an individual plant transpires have been a major limitation to breeding for transpiration efficiency. This has resulted in the selection for drought tolerance rather than applying a direct selection for water use (Cooper *et al.* 2014; Gaffney *et al.* 2015). One alternative method is the use of leaf stable carbon isotopes as a proxy for transpiration efficiency. The stable carbon isotope composition, δ^13^C, reflects the amount of ^13^C present in plant tissue relative to a standard (Keeling 1979). Enzymes in the process of carbon fixation discriminate differently against the heavier ^13^C atoms in a process known as fractionation (Farquhar *et al.* 1982; O’Leary 1988). It has been widely shown that stable carbon isotopes can be used as a proxy trait for quantifying a plant’s transpiration efficiency in C_3_ plants (Farquhar *et al.* 1989a,1989b; Condon *et al.* 1990; Virgona *et al.* 1990; Condon *et al.* 1993; Barbour *et al.* 2010) and in C_4_ plants (Henderson *et al.* 1998; von Caemmerer *et al.* 2014; Ellsworth *et al.*^2017^, ^2020^; Twohey III *et al.* 2019). Due to the differences in the photosynthetic pathways, δ^13^C is positively correlated with WUE in C_3_ species, but negatively correlated with δ^13^C in C_4_ species. Studies have also shown that δ^13^C can be influenced by environmental factors such as light intensity and drought, as well as anatomical traits and bundle‐sheath leakiness to CO_2_ (reviewed in Cernusak *et al.* 2013). However, genes underlying variation for δ^13^C remains unknown in C_4_ species.

Kolbe *et al.* (2018b) showed that leaf δ^13^C did not correlate with the leaf activity of any of the photosynthetic enzymes previously posited to influence δ^13^C variation. In addition, a transcriptome analysis was unable to identify a clear candidate gene (Kolbe *et al.* 2018b). Quantitative genetic approaches have the potential to reveal the genetic control of δ^13^C in C_4_ species because genomic locations are tested for associations with the trait of interest, without *a priori* knowledge of the mechanism underlying the variation. Maize is ideal for use in mapping studies due to its high level of recombination and low linkage disequilibrium (Yu and Buckler 2006). Mapping methods have been successfully used for decades to identify genes controlling complex traits in maize, with evolving approaches to tackle more difficult traits (Wallace *et al.* 2014). In addition, maize is both a model organism with available populations and genomic data, and one of the three most important global crops contributing to 30% of the total calories consumed by humans (Shiferaw *et al.* 2011).

There have been several previous studies that have used quantitative genetics to investigate δ^13^C in C_3_ species (Teulat *et al.* 2002; Masle *et al.* 2005; Rebetzke *et al.* 2008; Xu *et al.* 2009). In Arabidopsis, the gene *ERECTA* was identified in a QTL study for isotopic discrimination and was found to alter transpiration efficiency by altering stomatal density (Masle *et al.* 2005). Genetic mapping of leaf δ^13^C has also been performed in the C_4_ species *Setaria viridis* (Feldman *et al.* 2018; Ellsworth *et al.* 2020) and kernel δ^13^C has been mapped in the C_4_ maize (Gresset *et al.* 2014; Avramova *et al.* 2019). Although the QTL found for C_4_ species still require fine-mapping to identify the causative gene, no correlation was observed between kernel δ^13^C and leaf δ^13^C (Foley 2012). The lack of correlation may be the result of post-photosynthetic fractionation (Badeck *et al.* 2005; Cernusak *et al.* 2013), and therefore mapping QTL for δ^13^C in leaves may reveal additional loci not found using kernels. In this study, we focus on leaf δ^13^C in maize and its association with leaf elemental composition. We leverage results from previous studies to select biparental mapping populations. Specifically, the NAM founder lines CML103, CML333, and Tx303 consistently contrast B73 with respect to leaf δ^13^C (Kolbe *et al.* 2018b; Twohey III *et al.* 2019). The founder line NC358 had a moderate leaf δ^13^C value (Kolbe *et al.* 2018b; Twohey III *et al.* 2019) and was also included in this study. In addition to leaf δ^13^C, we also investigated variation in specific leaf area (SLA) and its potential relationship to leaf δ^13^C by CO_2_ diffusion. Characterization of the genetic architecture of leaf δ^13^C will provide a better context for understanding what drives δ^13^C, which will allow breeders to utilize this trait in crop improvement.

## Materials and methods

### Plant material

All experiments were planted at the University of Illinois Crop Sciences Research Farm, Urbana, IL, USA and were subject to natural conditions without supplemental irrigation. Plants did not show signs of stress prior to sample collection. Weather data collected less than 1.5 km from the field site can be found in Supplementary Table S5 (Water and Atmospheric Resources Monitoring Program, Illinois Climate Network 2020). NAM RIL families CML103, CML333, NC358, and Tx303 and NAM founder parents (McMullen *et al.* 2009) are publicly available through the Maize Genetic Cooperative Stock Center. The NAM RIL families were planted in the summer of 2015 and a subset was planted in 2019 using an augmented incomplete block design. Fifteen kernels were planted in each 3.7 m row with 0.8 m spacing between rows and 0.9 m alleys. The families were randomized together, with each block consisting of 20 lines and 2 checks (B73 and one of the other founder lines). Ten percent of the plots were dedicated to checks with the common parent B73 appearing in each block with one of the four founder lines. All lines used for the GWAS experiment are publicly available through the USDA Germplasm Resources Information Network (GRIN). This experiment was planted in the summer of 2016. Twenty kernels were planted in each 3.7 m row with 0.8 m spacing between rows and 0.9 m alleys, and then thinned to 15 plants per row. The reference line B73 was replicated 22 times within the experiment. A complete list of lines used can be found in Supplementary Tables S1–S3.

### Tissue sampling

Samples for δ^13^C analysis from the 2015 NAM RIL populations were collected 6 weeks after planting (approximately V9) as follows. A rectangular piece of tissue approximately 7.5 cm × 5 cm was taken from the middle of the leaf blade (excluding the midrib) of the uppermost fully expanded leaf from four plants in each plot. Samples were placed in a coin envelope and dried at 65°C for at least 7 days. After drying, four-hole punches (each 0.058532 cm^2^) were taken and placed in a 6 mm × 4 mm tin capsules (OEA Laboratories # C11350.500P) for analysis using a Delta PlusXP (Washington State University) isotope ratio mass spectrometer. Leaf samples for SLA measurements were collected from four plants in each of the plots (preferentially but not necessarily the same plants as were collected for δ^13^C) using a 1.6 cm diameter cork borer. Leaf discs were dried at 65°C for at least 7 days prior to weighing on an analytical balance (Model MS204S). SLA was calculated as the area of a leaf disc divided by its dry weight. These same leaf discs were then used for ionomics analyses as described in Pauli *et al.* (2018). Leaf samples for δ^13^C analysis from the GWAS panel and the 2019 NAM RIL population were collected at approximately V10 (6–7 weeks after planting) using the hole punch method and processed as described previously (Twohey III *et al.* 2019). Due to the high level of diversity in this panel, some lines were flowering when samples were collected, which resulted in tissue being collected from the flag leaf. These samples were analyzed using a Costech instruments elemental combustion system and a Delta V Advantage isotope ratio mass spectrometer.

### Statistical analysis

All analyses were completed using custom scripts (available upon request) and statistical packages in R (R Core Team 2017). A mixed model approach was utilized to obtain the best linear unbiased estimation (BLUE) of the fixed genotypic effect (Henderson 1975). For both years 2015 and 2019, the experimental design of the RIL panel was blocked containing partial repeated entries of the parental genotypes, B73 and CML103. A mixed-effect model was created using the R package “lme4” to account for the variation among repeated entries and to estimate δ^13^C for each genotype (Bates *et al.* 2015). In the model, genotypes were treated as fixed effects with year and block terms treated as random effects. In addition, the block term was nested within year as the genotypes were randomly assigned to blocks each year. The R package “emmeans” utilized Least-Square Means to provide BLUEs for each genotype (Searl *et* *al.* 1980).

#### Correlation analysis

Pearson correlations using phenotype mean values were calculated with corr.test() in R package “psych” (Revelle 2017) using complete observations and Holm’s method (Holm 1979) to adjust *P*-values for multiple testing. The correlation matrix was visualized using pairs.panel() in the R package “psych” (Revelle 2017).

#### Single family QTL mapping

The analysis was completed using NAM_phasedImputed_1cM_AllZeaGBSv2.3 dataset. The file contains fully imputed and phased genotypes for most of the RILs in the NAM population (Zhao *et al.* 2006; Lipka *et al.* 2015). This HapMap format file was converted to numeric format where 0 is the B73 homozygote reference, 1 is a heterozygote, and 2 is the homozygote alternative parent. Phenotypic means were regressed onto genotype. Lowest *P*-values from the ANOVA values of the linear model were recorded (*i.e.*, pvalues[i] = anova(lm(mypheno∼geno[i , ])). The previously identified marker was added to the model and re-run in a stepwise regression procedure. The final model included all identified QTL. Significance thresholds were determined by 200 permutations and alpha was set at 0.05. All analysis was completed using custom scripts in R (R Core Team 2017). Results were then compared to composite interval QTL mapping completed in R package “r/QTL” (Broman *et al.* 2003). QTL locations are indicated using B73 version 2 positions.

#### Joint linkage mapping

The analysis was completed using HapMapv2 (Chia *et al.* 2012). The genotypic dataset consisted of 836 markers were scored on 624 RILs from four biparental families with B73 as a common parent. The marker subset is composed of markers that could be placed unambiguously on the physical map, previously described in Brown *et al.* (2011). Unambiguous markers are defined by those anchored in CDS positions of genes that have held consistent over genome versions verified by MaizeGDB cross-reference tables. The markers are approximately evenly spaced across the genome with an average spacing of 1.6 cM. Missing data were imputed as previously described in Tian *et al.* (2011). Joint linkage models were constructed using custom script in R (R Core Team 2017) by a stepwise regression procedure. In general, we used linkage to test every marker across all four families to find the most significant QTL. The model has a family term and a marker: family term. The family term accounts for differences in mean phenotype between families. Inclusion of the marker: family term means that for each QTL we are assigning a separate effect to each family. The family term was included in the model and each of the 836 possible marker-by-family terms were assessed following the method of Ogut *et al*. (2015). The lowest *P*-values from the ANOVA values of the linear regression model were recorded (*i.e.*, JL_pvalues[i] = anova(lm(my_pheno∼family+geno[, i]: family)). All 836 marker-by-family terms were tested. SNP effects were nested in families to reflect the potential for unique QTL allele effects within each family. Significance thresholds were determined using 1000 permutations for each family independently and alpha was set at 0.05. The lowest resulting *P*-value was recorded for each permutation.

#### Genome-wide association study

A subset of 413 of 503 diverse lines from Hirsch *et al.* (2014) that included the Wisconsin Diversity Set of Hansey *et al.* (2011) was grown in 2016 and listed in Supplementary Table S2. Hirsch *et al.* (2014) collected RNA from whole seedling tissue which was sequenced via IlluminaHiSeq and filtered to create a working set of 485,179 SNPs that is available at https://datadryad.org//resource/doi : 10.5061/dryad.r73c5. The 413 lines were grown in 2016 and tissue was sampled when B73 was at the developmental stage V10. Isotopic analysis is described above. A genome-wide association analysis was run using R package “GAPIT” (Lipka *et al.* 2012) on leaf δ^13^C. Removal of SNPs with a minor allele frequency of less than 0.05 resulted in a subset of 438,222 SNPs being used in this analysis. A MLM model was used with model selection set to true to find the optimum number of principal components to account for population structure (Lipka *et al.* 2012). Significance thresholds were calculated using the Bonferroni correction of familywise error rate. An alternative significance test was calculated using the Benjamini-Hochberg procedure for controlling the false discovery rate (Benjamini and Hochberg 1995).

### Data availability

Genotypic datasets were downloaded from Panzea CyVerse iPlant Data Storage Commons (http://datacommons.cyverse.org/browse/iplant/home/shared/panzea). All phenotypic datasets were quality controlled for complete technical replicates and availability of genotypic data. A list of all genotypes used in each analysis is provided in Supplementary Tables S1–S4 and have been uploaded to figshare. Briefly, the δ^13^C analysis was completed with 640 RILs; including 156 CML103 RILs, 160 CML333 RILs, 159 NC358 RILs, and 165 Tx303 RILs (Supplementary Table S1). The element analysis was completed using a total of 704 RILs; including 175 CML103 RILs, 181 CML333 RILs, 175 NC358 RILs, and 173 Tx303 RILs (Supplementary Table S1). The SLA analysis used a total of 683 RILs; including 172 CML103 RILs, 176 CML333 RILs, 168 NC358 RILs, and 167 Tx303 RILs (Supplementary Table S1). The Joint linkage analysis was completed using a total of 624 RILs; including 154 CML103 RILs, 159 CML333 RILs, 151 NC358 RILs, and 160 Tx303 RILs (Supplementary Table S2). Supplementary Table S3 lists the 413 lines used in the GWAS of leaf δ^13^C. Supplementary Table S4 includes the QTL coordinates identified in the elemental QTL analyses. Supplementary Figure S1 shows the distribution of leaf δ^13^C for each of the NAM RIL families. Supplementary Figure S2 presents the correlation matrix for the elemental analysis, and Supplementary Figure S3 shows the chromosomes where significant QTL were identified for each element. Supplementary Figure S4 is the LOD plot from the GWAS mapping of leaf δ^13^C. Supplementary material is available at figshare: https://doi.org/10.25387/g3.14114234.

## Results

### Single-family QTL mapping

The RIL families generated from four NAM founder lines (CML103, CML333, NC358, and Tx303) were grown for linkage analysis. Consistent with previous studies, both the CML103 and CML333 parent lines had a significantly less negative leaf δ^13^C than B73 (*P *<* *0.05), when grown as replicated controls among the RILs. However, the Tx303 and NC358 parental lines were not found to be significantly different from B73. Transgressive segregation was observed in all four RIL families (Supplementary Figure S1).

**Figure 1:**
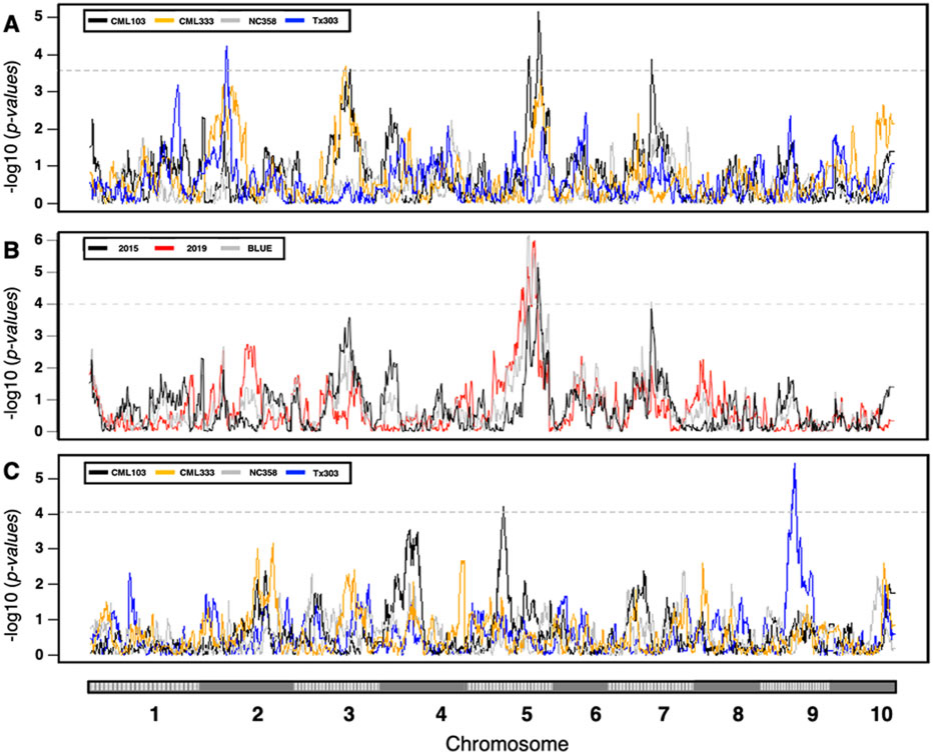
δ^13^C and SLA Single Family Stepwise Regression QTL Mapping. δ^13^C QTL (A) were identified in NAM RIL families CML103 (black), CML333 (orange), and Tx303 (blue) but not in NC358 (gray). Panel (B) shows the CML103 RIL family QTL from 2015 (black), 2019 (red), and the combined analysis (gray). SLA QTL (C) were identified in NAM RIL families CML103 (black) and Tx303 (blue). Significance thresholds (dashed horizontal line) were determined by 200 permutations and an alpha of 0.05.

Stepwise regression analyses found significant QTL for leaf δ^13^C in the NAM RIL families CML103, CML333, and Tx303 but not in NC358 (Figure 1A). Interestingly, none of these QTL were shared between RIL families in this analysis. The CML103 RIL family had two significant QTL, one on chromosome 5 at 211.7 Mb and another on chromosome 7 at 142.4 Mb. Combined these two QTL accounted for 21.36% of the total phenotypic variance (*R*^2^ = 0.2136; Table 1). The RIL family CML333 had one significant QTL on chromosome 3 at 183.9 Mb, which accounted for 8.37% of the total phenotypic variation explained (Table 1). Finally, the Tx303 RIL family had a significant QTL on chromosome 2 at 13.5 Mb explaining 9.48% of phenotypic variation (Table 1). No significant QTL for leaf δ^13^C were identified in the NC358 RIL family.

**Table 1:** δ^13^C single family stepwise regression QTL

Year	Step	Marker	Family	Chr.	Peak position (Mb)	*P*-value	**TPVE** ^a^ **(%)**	Effect size	1 LOD interval (Mb)
2015	1	824	CML103	5	211.7	7.03E−06	12.32	0.1286	211.5–212.9
2015	2	1032	CML103	7	142.4	7.52E−05	9.08	−0.1064	141.2–149.7
2015	1	470	CML333	3	183.9	2.07E−04	8.37	0.1137	178.6–195.7
2015	1	251	Tx303	2	13.5	6.04E−05	9.48	−0.1457	13.5–15.2
2019	1	818	CML103	5	210.8	1.00E−06	13.46	0.0927	208.2–210.4
BLUE	1	805	CML103	5	205.5	7.86E−07	13.26	0.0958	204.8–208.0

a Total percent of variation explained.

SLA was used as a proxy trait to test for a relationship between leaf thickness and leaf δ^13^C. No significant correlation was observed between SLA and leaf δ^13^C (*P *=* *0.304, *r* = −0.0414). In addition to testing for a correlation with leaf δ^13^C, QTL mapping was performed for SLA to identify any possible overlaps with genomic regions identified for leaf δ^13^C. Mapping of SLA in the four RIL families identified two significant QTL. In the CML103 RIL family, a QTL was identified on chromosome 5 at 86.1 Mb and in the Tx303 RIL family a QTL on chromosome 9 at 107.8 Mb. Neither of the SLA QTL identified overlapped with QTL for leaf δ^13^C (Figure 1C).

**Figure 2:**
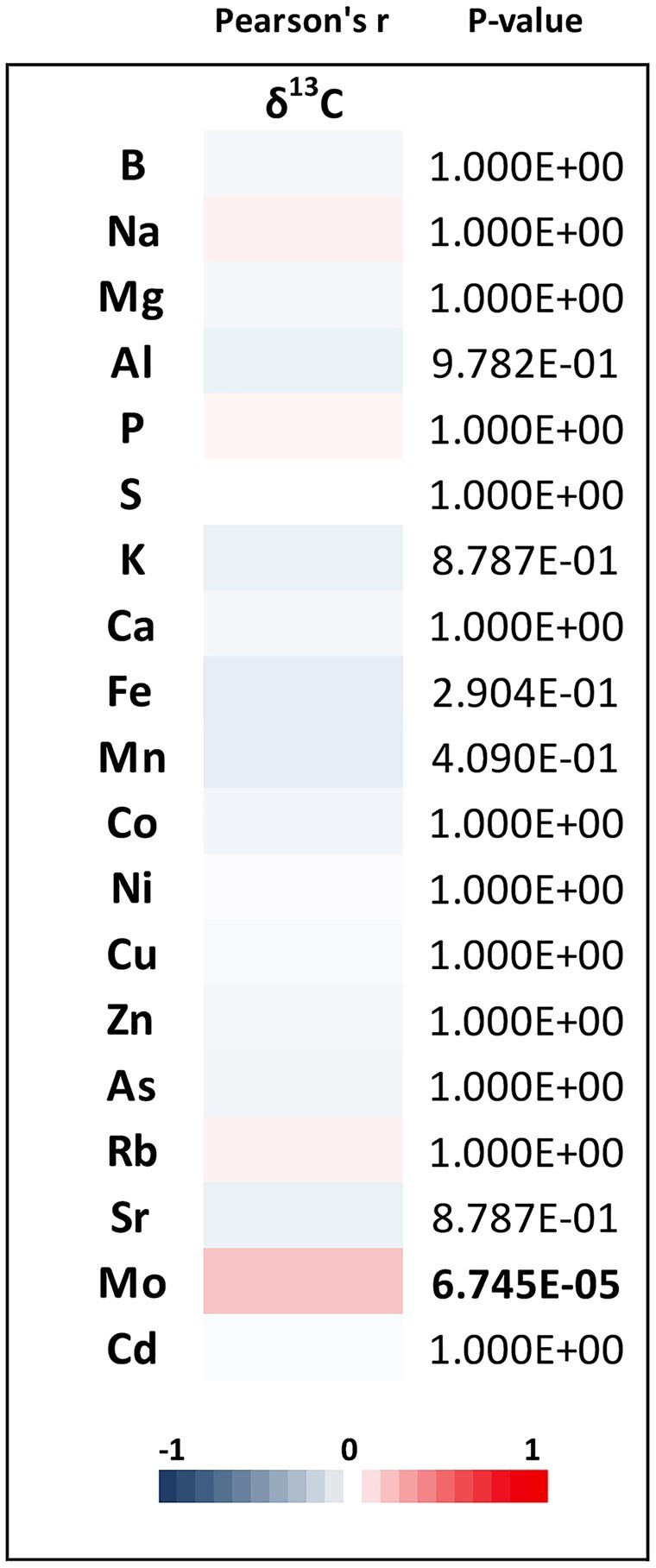
Pearson’s *r* Correlations. Correlations of mean phenotypic values using complete observations and Holm’s method to adjust *P*-values for multiple testing.

**Figure 3:**
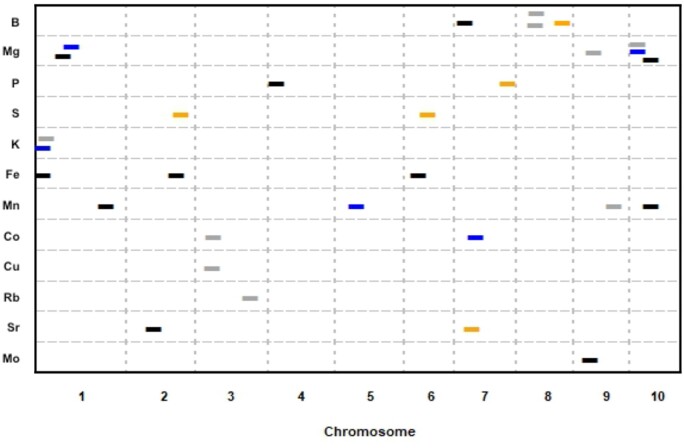
Element Single Family Stepwise Regression QTL Mapping. QTL mapping identified 28 QTL across 12 different elements. Significant QTL (alpha = 0.05) for each element are plotted. QTL location is shown across the 10 maize chromosomes (cM) on the *x*-axis. Dashes indicate a significant QTL, with the NAM RIL family in which the QTL was found designated by color; CML103 (black), CML333 (orange), Tx303 (gray), NC358 (blue). All dashes are the same length for visibility.

To test a potential link between transpiration and nutrient uptake, an elemental analysis was performed on leaf samples from each of the four RIL families. Samples were analyzed for 19 different elements using an ICP-MS. A full correlation matrix shows that some elements are highly correlated with each other (Supplementary Figure S2), but no strong correlations (*r* > ± 0.7) were identified with δ^13^C. However, there was a weak but significant correlation (*P *=* *6.745E-05, *r* = 0.18) between leaf δ^13^C and Mo (Figure 2). Subsequent QTL mapping of the 19 element concentrations identified 28 QTL across 12 different elements (Figure 3, Supplementary Figure S3). Significant QTL were found for B, Mg, P, S, K, Fe, Mn, Co, Cu, Rb, Sr, and Mo (Supplementary Table S4). None of the elemental QTL overlapped with those found for leaf δ^13^C or SLA. However, in some cases multiple elements had common QTL, such as Mg and Mn on chromosome 10 in the CML103 RIL family and Co and Cu on chromosome 3 in the NC358 RIL family. In addition, common QTL for an element were found across families, as in the case of Mg in the NC358 and Tx303 RIL families.

### Joint linkage QTL mapping

A joint linkage analysis was performed for leaf δ^13^C to test whether any additional QTL would be identified by combining the four RIL families into a single analysis. The joint linkage analysis identified the same significant QTL for leaf δ^13^C on chromosomes 2, 3, and 5 (Table 2) as in the single-family stepwise regression analysis. Although the QTL on chromosome 7 was not found using the joint linkage approach, an additional significant QTL was identified on chromosome 1. Given that no significant QTL for leaf δ^13^C were identified in the NC358 RIL family, we tested whether removing this family from the joint linkage analysis would change the outcome. When the joint linkage analysis was rerun excluding family NC358, the same four QTL were reidentified with decreased *P*-values, and the total phenotypic variation explained (*R*^2^ value) increased in later steps of the model. However, no new QTL was identified with this approach.

**Table 2:** δ^13^C joint linkage mapping QTL

Step	Marker	Family	Chr.	Peak position (Mb)	*P*-value	TPVE^a^ (%)	Effect size	1 LOD interval (Mb)
All four families								
1	m0200	Tx303	2	13.8	2.82E−06	7.9	−0.144	12.6–15.8
2	m0677	CML103	5	211.2	1.98E−04	11.8	0.123	211.2–212.7
3	m0385	CML103	3	182.1	1.44E−04	15.24	0.115	180.0–195.3
4	m0132	Tx303	1	263.2	4.41E−05	18.19	−0.126	257.1–263.6
Excluding NC358								
1	m0200	Tx303	2	13.8	5.70E−06	7.47	−0.144	12.6–15.9
2	m0677	CML103	5	211.2	2.86E−04	12.31	0.123	211.2–212.7
3	m0385	CML103	3	182.1	1.60E−04	16.45	0.115	180.0–195.3
4	m0132	Tx303	1	263.2	5.97E−05	20.05	−0.121	253.0–263.6

### QTL are consistent across years

One of the biparental mapping populations was grown in a second field trial to investigate the repeatability of the QTL between years. The CML103 RIL family had a significant QTL on chromosome 5 in both years (Figure 1B). The peak of the QTL shifted slightly (<1 Mb) between years, such that the 1 LOD intervals did not overlap (Table 1). However, there was overlap when a 2 LOD interval was calculated. A Best Linear Unbiased Estimate (BLUE) was calculated using the blocking information to account for spatial effects in each grow out. Again a QTL was identified in the same region, although shifted from what was observed when analyzing each year independently. The second QTL observed on chromosome 7 in the 2015 CML103 RIL family was not significant in 2019, nor was it significant when using BLUE for mapping.

### Genome-wide association study

Once significant QTL intervals were identified for leaf δ^13^C using a biparental mapping strategy (Figure 1, A and C), we performed a genome-wide association study to try and narrow down the intervals to specific genic regions. The Wisconsin Diversity Panel was chosen because it represents a large portion of variation found within maize and has a robust publicly available 485,179 SNP set. A subset of 413 of the possible lines was chosen due to seed availability, and were grown in a single randomized block. No significant SNP associations with leaf δ^13^C were identified (Supplementary Figure S4).

## Discussion

Leaf δ^13^C has a moderately high heritability in maize (Twohey III *et al.* 2019), which facilitates the use of quantitative genetics approaches to pinpoint the genomic locations controlling this trait. Here we characterized the genetic control of δ^13^C in maize using leaf tissue collected at vegetative stage V9-V10 to reflect the photosynthetic pool during active growth. We were able to identify several significant QTL for leaf δ^13^C across three NAM RIL families using stepwise regression. Using these populations we were also successful in identifying QTL for SLA and 12 different elements. Contrary to our hypothesis, no significant correlation was observed between leaf δ^13^C and SLA or elemental composition.

We strategically picked NAM RIL families based on the founder parents that had the largest differences in their δ^13^C for single family and joint linkage analyses. However, we also included a parent which was not extremely different from B73. Interestingly, we were unable to identify significant QTL in the NC358 RIL family despite transgressive segregation. We interpret this result as an indication the NC358 contains only small effect QTL relative to B73 that were not detected in this study. Alternatively, NC358 leaf δ^13^C may be more sensitive to the growing environment with a smaller genetic component. Twohey III *et al.* (2019) noted that while several maize lines were stable when tested in greenhouse and field environments, there were other lines that had highly variable isotopic signatures. A large amount of environmental influence over this trait in some backgrounds would obscure the genetic contribution and our ability to detect significant QTL.

When we compared the regions identified here with regions previously mapped in *S. viridis* no obvious overlap was observed (Ellsworth *et al.*, 2020). However, of the QTL identified for kernel δ^13^C in maize (Gresset *et al.* 2014; Avramova *et al* 2019), our QTL for leaf δ^13^C overlapped those on chromosomes 1, 3, and 7. This result demonstrates that some QTL for δ^13^C may be shared between tissues, and that these QTL are identified across several populations and environments. Indeed, when the CML103 RIL family was grown over two seasons the major effect QTL on chromosome 5 was observed in both growing environments.

Although the QTL analyses presented here do not provide gene-level resolution, we were able to look for candidate genes within the intervals. The 2015 chromosome 5 QTL includes an Erecta-like gene (*er1*, GRMZM2G463904, 211.8 Mb). However, *er1* was located outside of the 1 LOD interval of the 2019 chromosome 5 QTL. Furthermore, mapping of BLUE further shifted the chromosome 5 QTL peak away from *er1* (Table 1). Unfortunately, stomatal density data were not collected on these populations, which would further support the role of *er1* in variation of leaf δ^13^C. This would be an interesting area of future research given that this gene was found to effect δ^13^C in Arabidopsis by changing stomatal density (Masle *et al.* 2005). We also looked for genes that have been previously shown to directly influence transpiration efficiency in maize (reviewed in Leakey 2019). However, none of these were found to be located in our QTL intervals.

The linkage analyses using biparental mapping populations identified several significant QTL, but none of the single-family QTL were independently identified in more than one family (Figure 1). This result indicates that leaf δ^13^C can be controlled by different factors depending on the genetic background. Furthermore, if in fact leaf δ^13^C is controlled by many small-effect QTL, this may explain why the GWAS did not identify any significant SNP associations with leaf δ^13^C. Identifying rare alleles with small to moderate effect size is a known weakness of the GWAS method (Bazakos *et al.* 2017). A better understanding of the mechanisms influencing leaf δ^13^C would allow future analyses to move beyond single marker tests and instead look at SNPs in genes representing a particular pathway or process that could be collectively significant. This approach was successfully used to study maize lipid biosynthesis (Li *et al.* 2019a).

The diffusion of CO_2_ into mesophyll cells is a potential source of variation in leaf δ^13^C, which could be linked to stomatal density or leaf thickness. Previous work in maize has shown that stomatal density is not correlated with leaf δ^13^C in a small diversity panel of maize (Foley 2012). SLA has not been linked to δ^13^C in maize, but in rice δ^13^C and SLA have shared QTL (This *et al.* 2010). In this study, we were able to test SLA and δ^13^C in four RIL families, and no correlation was observed. Likewise, a comparison of the QTL analyses showed no overlapping regions for SLA and those mapped for leaf δ^13^C. This result suggests two possibilities. First, it is possible that differences in SLA observed in these populations are not due to leaf thickness, but rather composition. Identifying the causative genes underlying the QTL would give insight into the mechanism. A second possibility is that leaf anatomical traits other than leaf thickness influence leaf δ^13^C. A variety of anatomical traits could affect δ^13^C and would not be directly captured by measurement of SLA, such as stomatal ratios, interveinal distance, mesophyll surface area, chloroplast placement, and cell wall thickness. Variation in anatomical traits has the potential to influence CO_2_ diffusion as well as bundle sheath leakiness to CO_2_, both of which could influence leaf δ^13^C.

With our data, we were able to indirectly test the relationship between nutrient uptake and transpiration. If reducing transpiration limits nutrient uptake, transpiration efficiency as a trait for increasing WUE would have limited application. The 19 elements tested here were previously reported to have narrow-sense heritabilities ranging from 0.11 to 0.66 (Baxter 2014). The only element found to be significantly correlated to leaf δ^13^C was Molybdenum. Molybdenum is required for several vital biological processes related to nitrogen and water (Baxter 2008). Because molybdenum is a required cofactor for ABA synthesis, maize plants overexpressing molybdenum cofactor sulfurase gene have increased drought tolerance (Lu *et al.* 2013). However, in our study, we observed a positive correlation between leaf δ^13^C and molybdenum, which is contrary to expectation given that an increase in δ^13^C signifies a decrease in WUE. Overall, it is encouraging that the majority of elements sampled were not associated with δ^13^C. This suggests that breeding for leaf δ^13^C as a means to reduce transpiration would be unlikely to result in plants with nutrient uptake deficiencies.

Although the main focus of this study was to investigate leaf δ^13^C and its relationship to SLA and nutrient accumulation, the QTL mapping of the analyzed elements was an interesting biproduct. Mapping the leaf ionome of the four RIL families resulted in many significant QTL, including some overlapping intervals for different elements. Multi element QTLs are common, and are thought to be due to loci affecting processes such as the acidification of the rhizosphere or altering the permeability of the casparian strip (Baxter 2015). Interestingly there was not much overlap between the ionomic QTL identified here and a previous study on kernels (Table 4; Baxter *et al.* 2014). The only overlapping QTL was for rubidium on chromosome 3. There are several possible causes for the limited overlap between these methods. There could be differences between the leaf and grain ionome due to differential mobilization of nutrients from vegetative tissues into kernels during grain fill. In addition, the ionome is strongly influenced by genotype by environment interactions, with many of the QTL identified in previous studies being location specific (Asaro *et al.* 2016). Environmental interactions may also explain the unexpected high correlation between Fe and Al. One possible mechanism for this correlation is the variance in soil pH, either due to field effects or to the acidification of the rhizosphere by the plants roots. Both Fe and Al are more available at lower pHs. Interestingly, the Fe QTL did overlap a Zinc and Iron transporter (ZmZIP5; Zm00001d036965) on chromosome 6 (Li *et al.* 2019b).

## References

[jkab222-B1] Adachi S , Yoshikawa K, Yamanouchi U, Tanabata T, Sun J, et al 2017. Fine mapping of carbon assimilation rate 8, a quantitative trait locus for flag leaf nitrogen content, stomatal conductance and photosynthesis in rice. Front Plant Sci. 8:60. 28197156 10.3389/fpls.2017.00060 PMC5282472

[jkab222-B2] Asaro A , Ziegler G, Ziyomo C, Hoekenga OA, Dilkes BP, et al 2016. The interaction of genotype and environment determines variation in the maize kernel ionome. G3 (Bethesda). 6:4175–4183. 27770027 10.1534/g3.116.034827 PMC5144985

[jkab222-B3] Avramova V , Meziane A, Bauer E, Blankenagel S, Eggels S, et al 2019. Carbon isotope composition, water use efficiency, and drought sensitivity are controlled by a common genomic segment in maize. Theor Appl Genet. 132:53–63. 30244394 10.1007/s00122-018-3193-4 PMC6320357

[jkab222-B4] Badeck F , Tcherkez G, Nogues S, Piel C, Ghashghaie J. 2005. Post‐photosynthetic fractionation of stable carbon isotopes between plant organs—a widespread phenomenon. Rapid Commun Mass Spectrom. 19:1381–1391. 15880634 10.1002/rcm.1912

[jkab222-B5] Barber SA. 1962. A diffusion and mass‐flow concept of soil nutrient availability. Soil Sci. 93:39–49.

[jkab222-B6] Barber SA , Walker JM, Vasey EH. 1963. Mechanisms for movement of plant nutrients from soil and fertilizer to plant root. J Agric Food Chem. 11:204–207.

[jkab222-B7] Barbour MM , Warren CR, Farquhar GD, Forrester G, Brown H. 2010. Variability in mesophyll conductance between barley genotypes, and effects on transpiration efficiency and carbon isotope discrimination. Plant Cell Environ. 33:1176–1185. 20199618 10.1111/j.1365-3040.2010.02138.x

[jkab222-B8] Bates D , Mächler M, Bolker B, Walker S. 2015. Fitting linear mixed-effects models using lme4. J Stat Soft. 67:1–48.

[jkab222-B9] Baxter I. 2009. Ionomics: studying the social network of mineral nutrients. Curr Opin Plant Biol. 12:381–386. 19481970 10.1016/j.pbi.2009.05.002 PMC2701637

[jkab222-B10] Baxter I. 2015. Should we treat the ionome as a combination of individual elements, or should we be deriving novel combined traits? J Exp Bot. 66:2127–2131. 25711709 10.1093/jxb/erv040 PMC4986723

[jkab222-B11] Baxter I , Muthukumar B, Park HC, Buchner P, Lahner B, et al 2008. Variation in molybdenum content across broadly distributed populations of *Arabidopsis thaliana* is controlled by a mitochondrial molybdenum transporter (MOT1). PLoS Genet. 4:e1000004. 18454190 10.1371/journal.pgen.1000004 PMC2265440

[jkab222-B12] Baxter IR , Ziegler G, Lahner B, Mickelbart MV, Foley R, et al 2014. Single-kernel ionomic profiles are highly heritable indicators of genetic and environmental influences on elemental accumulation in maize grain (*Zea mays*). PLoS One. 9:e87628. 24489944 10.1371/journal.pone.0087628 PMC3906179

[jkab222-B13] Bazakos C , Hanemian M, Trontin C, Jiménez-Gómez JM, Loudet O. 2017. New strategies and tools in quantitative genetics: how to go from the phenotype to the genotype. Annu Rev Plant Biol. 68:435–455. 28226236 10.1146/annurev-arplant-042916-040820

[jkab222-B471] Benjamini Y, Hochberg Y. 1995. Controlling the false discovery rate: a practical and powerful approach to multiple testing. Journal of the Royal statistical society: series B (Methodological), 57:289–300.

[jkab222-B14] Broman KW , Wu H, Sen Ś, Churchill GA. 2003. R/qtl: QTL mapping in experimental crosses. Bioinformatics. 19:889–890. 12724300 10.1093/bioinformatics/btg112

[jkab222-B15] Brown PJ , Upadyayula N, Mahone GS, Tian F, Bradbury PJ, et al 2011. Distinct genetic architectures for male and female inflorescence traits of maize. PLoS Genet. 7:e1002383. 22125498 10.1371/journal.pgen.1002383 PMC3219606

[jkab222-B16] Bunce JA. 2010. Leaf transpiration efficiency of some drought-resistant maize lines. Crop Sci. 50:1409–1413.

[jkab222-B17] Cernusak LA , Ubierna N, Winter K, Holtum JAM, Marshall JD, et al 2013. Environmental and physiological determinants of carbon isotope discrimination in terrestrial plants. New Phytol. 200:950–965. 23902460 10.1111/nph.12423

[jkab222-B18] Chapman SC , Chakraborty S, Dreccer MF, Howden SM. 2012. Plant adaptation to climate change—opportunities and priorities in breeding. Crop Pasture Sci. 63:251–268.

[jkab222-B19] Chaves MM , Pereira JS, Maroco J, Rodrigues ML, Ricardo CPP, et al 2002. How plants cope with water stress in the field? Photosynthesis and growth. Ann Bot. 89:907–916. 12102516 10.1093/aob/mcf105 PMC4233809

[jkab222-B20] Chia J , Song C, Bradbury PJ, Costich D, Leon ND, et al 2012. Maize HapMap2 identifies extant variation from a genome in flux. Nat Genet. 44:803–807. 22660545 10.1038/ng.2313

[jkab222-B21] Condon AG , Farquhar GD, Richards RA. 1990. Genotypic variation in carbon isotope discrimination and transpiration efficiency in wheat. Leaf gas exchange and whole plant studies. Funct Plant Biol. 17:9–22.

[jkab222-B22] Condon AG , Richards RA, Farquhar GD. 1993. Relationships between carbon isotope discrimination, water use efficiency and transpiration efficiency for dryland wheat. Aust J Agric Res. 44:1693–1711.

[jkab222-B23] Condon AG , Richards RA, Rebetzke GJ, Farquhar GD. 2004. Breeding for high water-use efficiency. J Exp Bot. 55:2447–2460. 15475373 10.1093/jxb/erh277

[jkab222-B24] Cooper M , Gho C, Leafgren R, Tang T, Messina C. 2014. Breeding drought-tolerant maize hybrids for the US corn-belt: discovery to product. J Exp Bot. 65:6191–6204. 24596174 10.1093/jxb/eru064

[jkab222-B25] Ellsworth P , Feldman M, Baxter I, Cousins A. 2020. A genetic link between whole-plant water use efficiency and leaf carbon isotope composition in the C_4_ grass *Setaria*. Plant J. 102:1234–1248. 10.1111/tpj.14696. 31968138

[jkab222-B26] Ellsworth PZ , Ellsworth PV, Cousins AB. 2017. Relationship of leaf oxygen and carbon isotopic composition with transpiration efficiency in the C_4_ grasses *Setaria viridis* and *Setaria italica*. J Exp Bot. 68:3513–3528. 28859378 10.1093/jxb/erx185 PMC5853516

[jkab222-B27] Farquhar GD , Ehleringer JR, Hubick KT. 1989a. Carbon isotope discrimination and photosynthesis. Annu Rev Plant Physiol Plant Mol Biol. 40:503–537.

[jkab222-B28] Farquhar GD , Hubick KT, Condon AG. 1989b. Carbon isotope fractionation and plant water-use efficiency. In:Rundel PW, Ehleringer JR, Nagy KA, Richards RA, editors. Stable Isotopes in Ecological Research. New York, NY: Springer. p. 21–40.

[jkab222-B271] Farquhar GD, O'Leary MH, Berry JA. 1982. On the relationship between carbon isotope discrimination and the intercellular carbon dioxide concentration in leaves. J Plant Physiol. 9:121–137.

[jkab222-B29] Feldman MJ , Ellsworth PZ, Fahlgren N, Gehan MA, Cousins AB, et al 2018. Components of water use efficiency have unique genetic signatures in the model C_4_ grass Setaria. Plant Physiol. 178:699–715. 30093527 10.1104/pp.18.00146 PMC6181048

[jkab222-B30] Flexas J , Díaz ‐Espejo A, Conesa MA, Coopman RE, Douthe C, et al 2016. Mesophyll conductance to CO_2_ and Rubisco as targets for improving intrinsic water use efficiency in C_3_ plants. Plant Cell Environ. 39:965–982. 26297108 10.1111/pce.12622

[jkab222-B371] Foley RC. 2012. The genetic diversity of water use efficiency in the nested associated mapping population of Zea mays (Doctoral dissertation, Purdue University).

[jkab222-B31] Gaffney J , Schussler J, Löffler C, Cai W, Paszkiewicz S, et al 2015. Industry‐scale evaluation of maize hybrids selected for increased yield in drought‐stress conditions of the US corn belt. Crop Sci. 55:1608–1618.

[jkab222-B32] Gresset S , Westermeier P, Rademacher S, Ouzunova M, Presterl T, et al 2014. Stable carbon isotope discrimination is under genetic control in the C4 species maize with several genomic regions influencing trait expression. Plant Physiol. 164:131–143. 24280436 10.1104/pp.113.224816 PMC3875796

[jkab222-B33] Hammer GL , Farquhar GD, Broad IJ. 1997. On the extent of genetic variation for transpiration efficiency in sorghum. Aust J Agric Res. 48:649–656.

[jkab222-B34] Hansey CN , Johnson JM, Sekhon RS, Kaeppler SM, de Leon N. 2011. Genetic diversity of a maize association population with restricted phenology. Crop Sci. 51:704–715.

[jkab222-B35] Henderson CR. 1975. Best linear unbiased estimation and prediction under a selection model. Biometrics. 31:423–447. 1174616

[jkab222-B36] Henderson S , Caemmerer SV, Farquhar GD, Wade L, Hammer G. 1998. Correlation between carbon isotope discrimination and transpiration efficiency in lines of the C_4_ species Sorghum bicolor in the glasshouse and the field. Funct Plant Biol. 25:111–123.

[jkab222-B37] Hirsch CN , Foerster JM, Johnson JM, Sekhon RS, Muttoni G, et al 2014. Insights into the maize pan-genome and pan-transcriptome. Plant Cell. 26:121–135. 24488960 10.1105/tpc.113.119982 PMC3963563

[jkab222-B38] Holm S. 1979. A simple sequentially rejective multiple test procedure. Scand J Stat. 6:65–70.

[jkab222-B39] Jaleel CA , Manivannan P, Wahid A, Farooq M, Al-Juburi HJ, et al 2009. Drought stress in plants: a review on morphological characteristics and pigments composition. Int J Agric Biol. 11:100–105.

[jkab222-B40] Keeling CD , Mook WG, Tans PP. 1979. Recent trends in the 13C/12C ratio of atmospheric carbon dioxide. Nature. 277:121–123.

[jkab222-B41] Kolbe AR , Brutnell TP, Cousins AB, Studer AJ. 2018a. Carbonic anhydrase mutants in *Zea mays* have altered stomatal responses to environmental signals. Plant Physiol. 177:980–989. 29794168 10.1104/pp.18.00176 PMC6053012

[jkab222-B42] Kolbe AR , Studer AJ, Cousins AB. 2018b. Biochemical and transcriptomic analysis of maize diversity to elucidate drivers of leaf carbon isotope composition. Funct Plant Biol. 45:489–500. 32290988 10.1071/FP17265

[jkab222-B43] Leakey ADB , Ferguson JN, Pignon CP, Wu A, Jin Z, et al 2019. Water use efficiency as a constraint and target for improving the resilience and productivity of C_3_ and C_4_ crops. Annu Rev Plant Biol. 70:781–808. 31035829 10.1146/annurev-arplant-042817-040305

[jkab222-B44] Li H , Thrash A, Tang JD, He L, Yan J, et al 2019a. Leveraging GWAS data to identify metabolic pathways and networks involved in maize lipid biosynthesis. Plant J. 98:853–863. 30742331 10.1111/tpj.14282 PMC6850169

[jkab222-B45] Li S , Liu X, Zhou X, Li T, Yang W, et al 2019b. Improving zinc and iron accumulation in maize grains using the zinc and iron transporter ZmZIP5. Plant Cell Physiol. 60:2077–2085. 31165152 10.1093/pcp/pcz104

[jkab222-B46] Lipka AE , Kandianis CB, Hudson ME, Yu J, Drnevich J, et al 2015. From association to prediction: statistical methods for the dissection and selection of complex traits in plants. Curr Opin Plant Biol. 24:110–118. 25795170 10.1016/j.pbi.2015.02.010

[jkab222-B47] Lipka AE , Tian F, Wang Q, Peiffer J, Li M, et al 2012. GAPIT: genome association and prediction integrated tool. Bioinformatics. 28:2397–2399. 22796960 10.1093/bioinformatics/bts444

[jkab222-B48] Lu Y , Li Y, Zhang J, Xiao Y, Yue Y, et al 2013. Overexpression of Arabidopsis molybdenum cofactor sulfurase gene confers drought tolerance in maize (*Zea mays* L.). PLoS One. 8:e52126. 23326325 10.1371/journal.pone.0052126 PMC3542365

[jkab222-B49] Marschner H , Dell B. 1994. Nutrient uptake in mycorrhizal symbiosis. Plant Soil. 159:89–102.

[jkab222-B50] Masle J , Farquhar GD, Wong SC. 1992. Transpiration ratio and plant mineral content are related among genotypes of a range of species. Funct Plant Biol. 19:709–721.

[jkab222-B51] Masle J , Gilmore SR, Farquhar GD. 2005. The ERECTA gene regulates plant transpiration efficiency in Arabidopsis. Nature. 436:866–870. 16007076 10.1038/nature03835

[jkab222-B52] McGrath JM , Lobell DB. 2013. Reduction of transpiration and altered nutrient allocation contribute to nutrient decline of crops grown in elevated CO_2_ concentrations. Plant Cell Environ. 36:697–705. 22943419 10.1111/pce.12007

[jkab222-B53] McMullen MD , Kresovich S, Villeda HS, Bradbury P, Li H, et al 2009. Genetic properties of the maize nested association mapping population. Science. 325:737–740. 19661427 10.1126/science.1174320

[jkab222-B54] Myers SS , Zanobetti A, Kloog I, Huybers P, Leakey AD, et al 2014. Increasing CO_2_ threatens human nutrition. Nature. 510:139–142. 24805231 10.1038/nature13179 PMC4810679

[jkab222-B55] National Academies of Sciences, Engineering, and Medicine 2018. Science Breakthroughs to Advance Food and Agricultural Research. Washington, DC: National Academic Press.

[jkab222-B56] O'Leary MH. 1988. Carbon isotopes in photosynthesis. Bioscience. 38:328–336.

[jkab222-B5377557] Ogut F , Bian Y, Bradbury PJ, Holland JB. 2015. Joint-multiple family linkage analysis predicts within-family variation better than single-family analysis of the maize nested association mapping population. Heredity (Edinb). 114:552–563. 25585918 10.1038/hdy.2014.123 PMC4434247

[jkab222-B57] Passioura JB. 1996. Drought and drought tolerance. Plant Growth Regul. 20:79–83.

[jkab222-B58] Pauli D , Ziegler G, Ren M, Jenks MA, Hunsaker DJ, et al 2018. Multivariate analysis of the cotton seed ionome reveals a shared genetic architecture. G3 (Bethesda). 8:1147–1160. 29437829 10.1534/g3.117.300479 PMC5873906

[jkab222-B59] Purcell LC , Edwards JT, Brye KR. 2007. Soybean yield and biomass responses to cumulative transpiration: Questioning widely held beliefs. Field Crops Res. 101:10–18.

[jkab222-B60] R Core Team 2017. R: A language and environment for statistical computing. R Foundation for Statistical Computing, Vienna, Austria. Available at: https://www.R-project.org/.

[jkab222-B61] Rebetzke GJ , Condon AG, Farquhar GD, Appels R, Richards RA. 2008. Quantitative trait loci for carbon isotope discrimination are repeatable across environments and wheat mapping populations. Theor Appl Genet. 118:123–137. 18818897 10.1007/s00122-008-0882-4

[jkab222-B62] Revelle WR. 2017. psych: Procedures for personality and psychological research. Software, https://CRAN.R-project.org/package=psych, version 1.8.4.

[jkab222-B63] Searle SR , Speed FM, Milliken GA. 1980. Population marginal means in the linear model: an alternative to least squares means. Am Stat. 34:216–221.

[jkab222-B64] Sheffield J , Wood EF. 2008. Projected changes in drought occurrence under future global warming from multi-model, multi-scenario, IPCC AR4 simulations. Clim Dyn. 31:79–105.

[jkab222-B65] Shiferaw B , Prasanna BM, Hellin J, Bänziger M. 2011. Crops that feed the world 6. Past successes and future challenges to the role played by maize in global food security. Food Sec. 3:307–327.

[jkab222-B66] Tian F , Bradbury PJ, Brown PJ, Hung H, Sun Q, et al 2011. Genome-wide association study of leaf architecture in the maize nested association mapping population. Nat Genet. 43:159–162. 21217756 10.1038/ng.746

[jkab222-B67] Teulat B , Merah O, Sirault X, Borries C, Waugh R, et al 2002. QTLs for grain carbon isotope discrimination in field-grown barley. Theor Appl Genet. 106:118–126. 12582879 10.1007/s00122-002-1028-8

[jkab222-B68] This D , Comstock J, Courtois B, Xu Y, Ahmadi N, et al 2010. Genetic analysis of water use efficiency in rice (*Oryza sativa* L.) at the leaf level. Rice. 3:72–86.

[jkab222-B69] Twohey RJ III , Roberts LM, Studer AJ. 2019. Leaf stable carbon isotope composition reflects transpiration efficiency in *Zea mays*. Plant J. 97:475–484. 30351458 10.1111/tpj.14135

[jkab222-B70] Virgona JM , Hubick KT, Rawson HM, Farquhar GD, Downes RW. 1990. Genotypic variation in transpiration efficiency, carbon-isotype discrimination and carbon allocation during early growth in sunflower. Functional Plant Biol. 17:207–214.

[jkab222-B71] von Caemmerer S , Ghannoum O, Pengelly JJL, Cousins AB. 2014. Carbon isotope discrimination as a tool to explore C_4_ photosynthesis. J Exp Bot. 65:3459–3470. 24711615 10.1093/jxb/eru127

[jkab222-B72] Walker GK. 1986. Transpiration efficiency of field-grown maize. Field Crops Res. 14:29–38.

[jkab222-B73] Wallace JG , Larsson SJ, Buckler ES. 2014. Entering the second century of maize quantitative genetics. Heredity (Edinb). 112:30–38. 23462502 10.1038/hdy.2013.6 PMC3860165

[jkab222-B74] Water and Atmospheric Resources Monitoring Program, Illinois Climate Network 2020. Illinois State Water Survey, 2204 Griffith Drive, Champaign, IL 61820-7495. 10.13012/J8MW2F2Q.

[jkab222-B75] Xu Y , This D, Pausch RC, Vonhof WM, Coburn JR, et al 2009. Leaf-level water use efficiency determined by carbon isotope discrimination in rice seedlings: genetic variation associated with population structure and QTL mapping. Theor Appl Genet. 118:1065–1081. 19224195 10.1007/s00122-009-0963-z

[jkab222-B76] Yu J , Buckler ES. 2006. Genetic association mapping and genome organization of maize. Curr Opin Biotechnol. 17:155–160. 16504497 10.1016/j.copbio.2006.02.003

[jkab222-B77] Zhao W , Canaran P, Jurkuta R, Fulton T, Glaubitz J, et al 2006. Panzea: a database and resource for molecular and functional diversity in the maize genome. Nucleic Acids Res. 34:D752–D757. 16381974 10.1093/nar/gkj011 PMC1347374

